# Supplementary material for: Association between oral microbiome and seven types of cancers in East Asian population: a two-sample Mendelian randomization analysis
Source: Front Mol Biosci. 2023 Nov 21;10:1327893. doi: 10.3389/fmolb.2023.1327893 (PMC10702768; doi:10.3389/fmolb.2023.1327893)
Supplement: Supplementary file 1 [file DataSheet1.ZIP › Supplementary Materials/MR plots of saliva/saliva═╝/colorectal cancer/pheno.2831_to_colorectal cancer_forest.pdf]

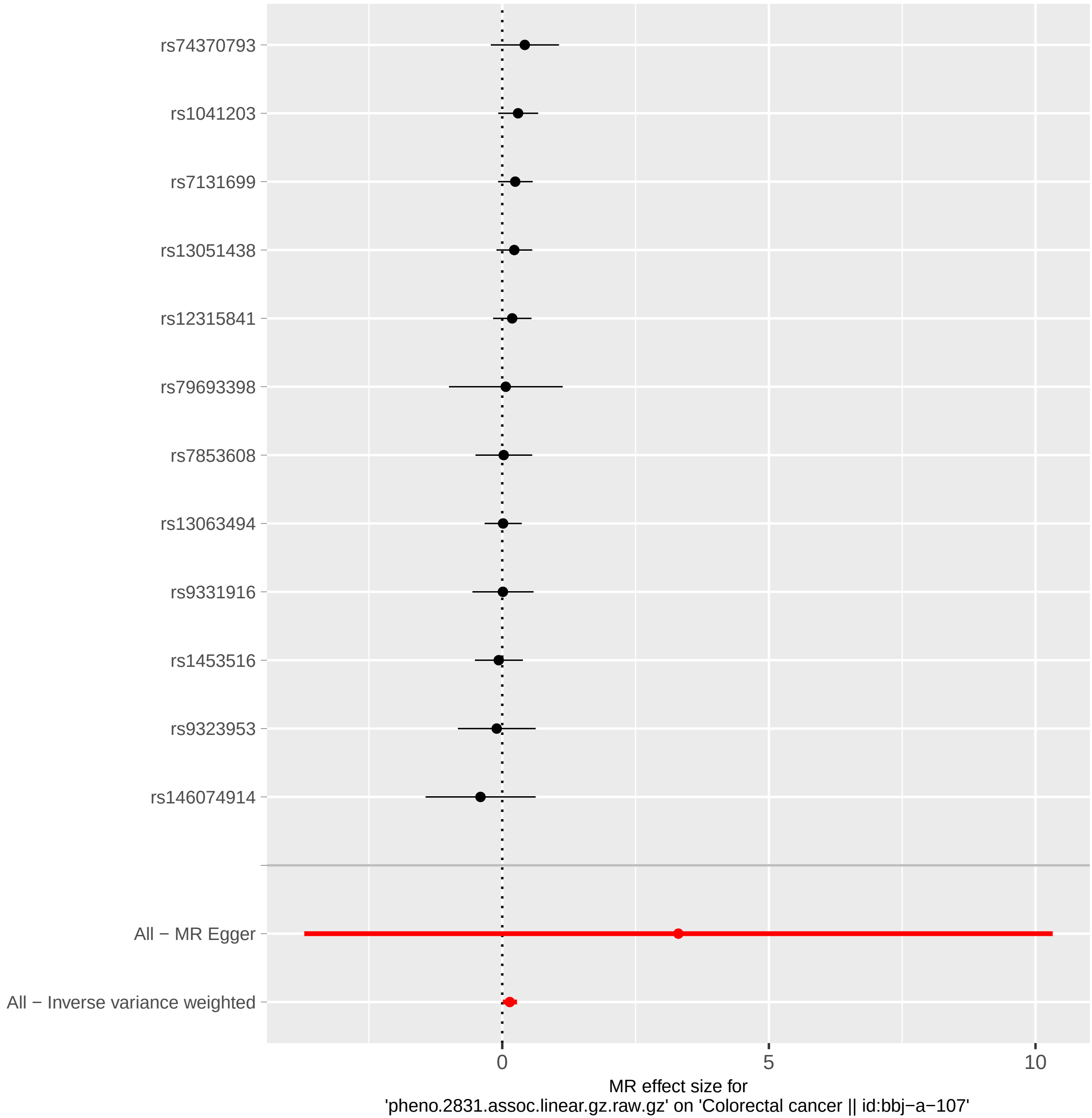
rs74370793
rs1041203
rs7131699
rs13051438
rs12315841
rs79693398
rs7853608
rs13063494
rs9331916
rs1453516
rs9323953
rs146074914
All − MR Egger
All − Inverse variance weighted
0
5
10
MR effect size for
'pheno.2831.assoc.linear.gz.raw.gz' on 'Colorectal cancer || id:bbj−a−107'
